# Autophagy restricts symbiosis-associated cell death and regulates colonization by *Serendipita indica* in Arabidopsis

**DOI:** 10.1093/plphys/kiaf590

**Published:** 2025-11-18

**Authors:** Patricia Zecua-Ramirez, Nick Dunken, Nyasha M Charura, Ernesto Llamas, Concetta De Quattro, Alexander Mandel, Gregor Langen, Yasin Dagdas, Alga Zuccaro

**Affiliations:** Institute for Plant Sciences, University of Cologne, Cologne, NW 50674, Germany; Institute for Plant Sciences, University of Cologne, Cologne, NW 50674, Germany; Institute for Plant Sciences, University of Cologne, Cologne, NW 50674, Germany; Institute for Plant Sciences, University of Cologne, Cologne, NW 50674, Germany; Cluster of Excellence on Plant Sciences (CEPLAS), Cologne, NW 50674, Germany; Institute for Plant Sciences, University of Cologne, Cologne, NW 50674, Germany; Cluster of Excellence on Plant Sciences (CEPLAS), Cologne, NW 50674, Germany; Institute for Plant Sciences, University of Cologne, Cologne, NW 50674, Germany; Institute for Plant Sciences, University of Cologne, Cologne, NW 50674, Germany; Centre for Organismal Studies (COS), Heidelberg University, Heidelberg, BW 69120, Germany; Institute for Plant Sciences, University of Cologne, Cologne, NW 50674, Germany; Cluster of Excellence on Plant Sciences (CEPLAS), Cologne, NW 50674, Germany

## Abstract

Endophytic colonization of Arabidopsis (*Arabidopsis thaliana*) by the beneficial root endophyte *Serendipita indica* is characterized by an initial biotrophic phase, followed by a confined host cell death phase that facilitates fungal accommodation. However, the host molecular pathways that restrict *S. indica* proliferation and regulate symbiosis-associated cell death remain largely unknown. Our study demonstrates that autophagy, a key cellular degradation pathway that maintains homeostasis, is locally activated during colonization and is required to limit fungal proliferation and immunometabolic stress. Autophagy-deficient mutants exhibit elevated basal root cell death, increased colonization, and hypersensitivity to the fungal-derived purine metabolite 2′-deoxyadenosine (dAdo), an immunometabolic signal that modulates host cell viability and reprograms immune and metabolic responses via ENT3 (equilibrative nucleoside transporter 3)-mediated uptake. In *ent3* and *atg5 ent3* mutants, suppression of dAdo import reduces *S. indica*-induced cell death, confirming the central role of ENT3-mediated uptake. Despite increased colonization and stress sensitivity, autophagy-deficient plants retain *S. indica*-mediated root growth promotion, indicating that mutualistic benefits can occur independently of immunometabolic stress resilience. Based on these findings, we propose that autophagy-mediated pro-survival responses are essential for maintaining symbiotic homeostasis by integrating immunometabolic signals and preserving host cell viability.

## Teaser

Autophagy acts as a pro-survival mechanism in plant roots during beneficial fungal colonization by restricting fungal proliferation and immunometabolic cell death triggered by a fungal metabolite.

## Introduction

Fungi that colonize plant roots employ diverse strategies to interact with their hosts, shaped by lifestyle and ecological niche (Lo Presti et al. 2015). Among beneficial endophytes, members of the Sebacinales (Basidiomycota), such as *Serendipita indica*, form mutualistic associations with a broad range of plant species, promoting growth and enhancing resistance to biotic and abiotic stresses (Oberwinkler et al. 2013; Weiss et al. 2016). Successful colonization by *S. indica* involves dynamic modulation of plant immunity, metabolism, and cell death pathways (Deshmukh et al. 2006; Jacobs et al. 2011; Nizam et al. 2019; Dunken et al. 2024). In Arabidopsis (*Arabidopsis thaliana*), *S. indica* exhibits a biphasic colonization pattern consisting of an initial biotrophic phase, characterized by intimate contact with living host cells, followed by a spatially confined cell death-associated phase (Deshmukh et al. 2006; Qiang et al. 2012). This latter phase occurs in specific root zones and enables the fungus to access nutrients and establish a symbiotic niche while contributing to improved plant growth and fitness (Zuccaro et al. 2011; Lahrmann et al. 2013; Weiss et al. 2016). Recent work has shown that *S. indica* secretes effector enzymes that convert extracellular DNA (eDNA) into 2′-deoxyadenosine (dAdo), a signaling nucleoside that, once imported into host cells via the nucleoside transporter ENT3 (equilibrative nucleoside transporter 3), triggers regulated cell death (Dunken et al. 2024). While this process appears to facilitate fungal accommodation, how the host limits or recovers from *S. indica*-triggered cell death remains unclear.

Autophagy, a highly conserved intracellular recycling pathway, essential for cellular homeostasis, plays key roles in plant stress adaptation, immune regulation, and the control of cell death (He and Klionsky 2009). Depending on the context, autophagy can either promote defense responses or suppress cell death to protect healthy tissues (Hofius et al. 2009; Yoshimoto et al. 2009; Lai et al. 2011; Munch et al. 2014; Han et al. 2015; Estrada-Navarrete et al. 2016). Although autophagy has been implicated in interactions with both pathogens and mutualists, its specific function during beneficial fungal colonization remains poorly defined.

In this study, we investigate how autophagy shapes the outcome of the *S. indica–A. thaliana* interaction. By analyzing *A. thaliana* mutants deficient in core autophagy genes (*atg5*, *atg2*, and *atg10*), we show that autophagy restricts fungal colonization and protects root cells from dAdo-induced cell death. Autophagy is locally activated at fungal contact sites, and its absence leads to enhanced stress sensitivity and impaired recovery following dAdo exposure. Strikingly, disruption of dAdo uptake via ENT3 in an autophagy-deficient background suppresses both dAdo- and symbiosis-mediated cell death, and partially limits fungal overgrowth. These findings suggest that autophagy mitigates immunometabolic stress downstream of dAdo uptake. Together, our results identify autophagy as a key regulator of root cell survival during symbiosis. By limiting dAdo-induced cell death and maintaining cellular integrity, autophagy enables the host to accommodate its fungal partner without compromising tissue health. This work reveals how plants fine-tune beneficial interactions through core stress resilience mechanisms.

## Results

### Autophagy is locally activated during *S. indica* colonization and restricts fungal proliferation

**Figure 1.**
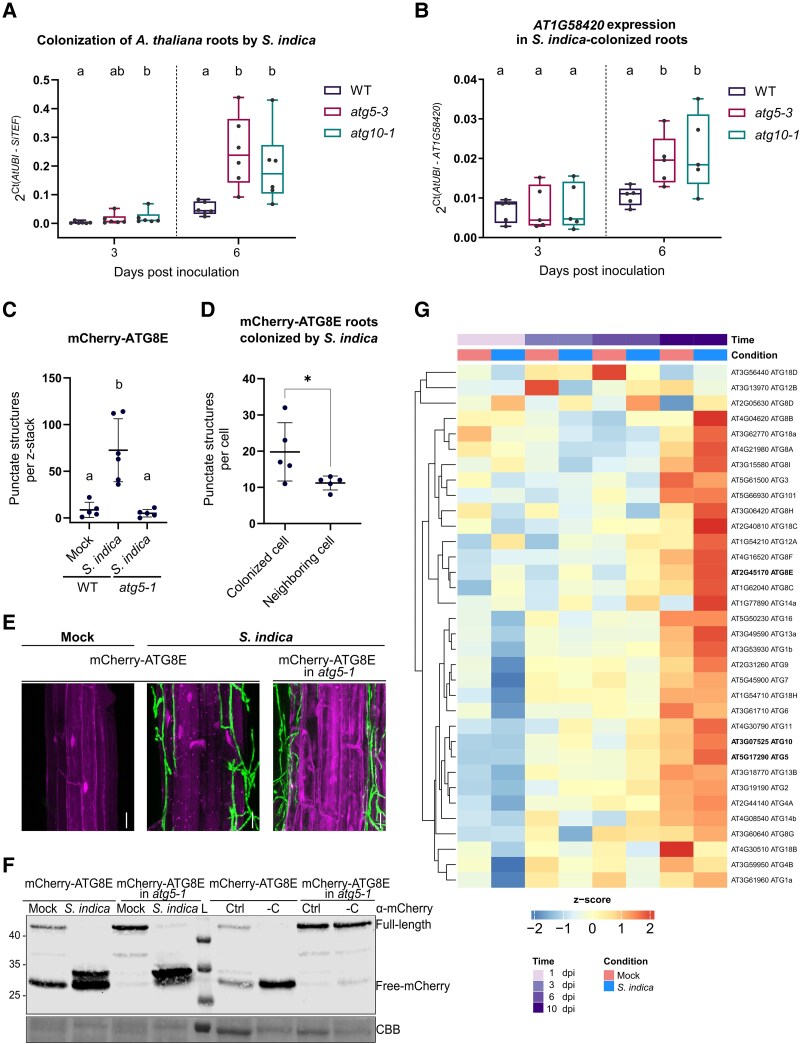
Involvement of autophagy in the colonization of *A. thaliana* roots by *S. indica*. **A)** Quantification of *S. indica* colonization in WT, *atg5-3*, and *atg10-1* roots at 3- and 6 dpi, measured by RT-qPCR. Fungal biomass was calculated as *SiTEF*/*AtUBI* transcript ratio using cDNA as template and the 2^−Δ*Ct*^ method. The box extends from the 25th to 75th percentiles, the center line is the median, and the whiskers extend to the minimum and maximum values. Data from 6 independent biological replicates. Different letters indicate statistically significant differences (*P* < 0.05; Kruskal–Wallis test with post hoc Dunn test and Benjamini–Hochberg correction). **B)** Expression of the *S. indica*-responsive marker gene *AT1G58420* in colonized WT, *atg5-3*, and *atg10-1* roots at 6 dpi. Expression values were normalized to *AtUBI* using the 2^−Δ*Ct*^ method. The box extends from the 25th to 75th percentiles, the center line is the median and the whiskers extend to the minimum and maximum values. Data are from 5 biological replicates. Different letters indicate statistically significant differences (*P* < 0.05; Kruskal–Wallis test with post hoc Dunn test and Benjamini–Hochberg correction). **C)** Quantification of mCherry-ATG8E-labeled puncta in differentiated root cells of transgenic plants expressing mCherry-ATG8E. Images were obtained by confocal microscopy from WT (mock or *S. indica*), and *atg5-1* background (*n* = 5–6) at 10 dpi. Dots represent the total number of puncta quantified from individual z-stack images (mean ± Sd). Different letters indicate statistically significant differences (*P* < 0.05; Kruskal–Wallis test with post hoc Dunn test and Benjamini–Hochberg correction). **D)** Localized quantification of mCherry-ATG8E puncta per root cell, comparing *S. indica*-contacted cells to adjacent, non-contacted cells. Dots represent the number of puncta quantified from individual root cells (mean ± Sd). Asterisk indicates significant difference (*P* < 0.05; unpaired Student's *t*-test; *n* = 5). **E)** Representative confocal laser scanning microscopy images of transgenic roots expressing mCherry-ATG8E in WT and *atg5-1* backgrounds following mock- or *S. indica* inoculation at 10 dpi. Images are shown as maximal projections of 8 to 10 optical sections. Scale bar: 20 *µ*m. **F)** Immunoblot analysis of autophagic flux in mCherry-ATG8E-expressing roots. Left: samples from roots treated with mock or *S. indica* at 10 dpi. Right: samples from seedlings incubated in control or carbon-depleted media. Blots were probed with anti-mCherry antibody; Coomassie brilliant blue was used as loading control. **G)** Heatmap showing expression of autophagy-related genes (ATGs) in WT roots at 1, 3, 6, and 10 dpi under *S. indica* or mock treatment. Genes with mean TPM > 1 (transcripts per million) across samples were included. *Z*-scores of log_2_(TPM + 1) values are displayed; data reflect the average of 3 biological replicates per condition. The full heatmap is available in Supplementary Fig. S2.

To investigate whether autophagy influences the outcome of *S. indica* colonization, we quantified fungal biomass in *A. thaliana* autophagy-deficient mutants *atg5-3* and *atg10-1* using RT-qPCR. These mutants are defective in autophagosome formation due to the disruption of the ATG12–ATG5 conjugation system, a central step in the core autophagy machinery (Thompson et al. 2005; Phillips et al. 2008). To ensure accurate quantification of internal colonization, root samples were thoroughly washed prior to RNA extraction to remove extraradical hyphae. At 6 d post inoculation (dpi), a time point coinciding with the onset of the cell death-associated phase, both *atg5-3* and *atg10-1* exhibited significantly higher levels of fungal colonization compared with wild-type (WT) Col-0 (Fig. 1A). Consistent with increased colonization, expression of *AT1G58420*, a known marker of *S. indica* colonization and general wound and stress signaling (Choi et al. 2014; Nizam et al. 2019; Yoo et al. 2020; Dunken et al. 2024), was significantly upregulated in autophagy mutants compared with WT (Fig. 1B). Immune-related genes *WRKY33* and *CYP81F2*, involved in defense activation and secondary metabolism (Bednarek et al. 2009; Clay et al. 2009; Birkenbihl et al. 2017), were also more strongly induced in *atg5-3* and *atg10-1* mutants (Supplementary Fig. S1), suggesting enhanced immune activity in response to elevated fungal load.

To determine whether autophagy is activated during colonization, we employed a fluorescent autophagy reporter line expressing pUbi::mCherry-ATG8E in WT and *atg5-1* backgrounds. Confocal imaging of the root maturation zone revealed a marked increase in mCherry-ATG8E-labeled puncta in colonized WT roots compared with mock controls (Fig. 1, C to E). Puncta were particularly enriched in cells directly contacting fungal hyphae, indicating localized autophagy activation at the host–fungus interface (Fig. 1D). In contrast, colonized *atg5-1* roots displayed fewer puncta and accumulated large mCherry-positive aggregates, consistent with impaired autophagic flux (Li et al. 2015).

We further validated autophagic activation using immunoblot analysis of mCherry-ATG8E. Colonized WT roots exhibited increased levels of free mCherry, a marker of active autophagic flux resulting from selective degradation of ATG8 (Chung et al. 2010; Fig. 1F). This response was absent in *atg5-1*, confirming that functional autophagy machinery is required. Interestingly, an additional cleavage fragment of mCherry-ATG8E was detected in both WT and *atg5-1* upon *S. indica* colonization, but not under carbon starvation, suggesting a colonization-specific cleavage mechanism independent of canonical ATG5-mediated autophagy, potentially mediated by host or fungal proteases active during the cell death-associated phase.

To complement these findings at the transcriptional level, we analyzed time-resolved transcriptomic data from colonized roots and found that the expression of several core *ATG* genes (Mizushima et al. 2011; Liu and Bassham 2012) increased during later stages of colonization (Fig. 1G; Supplementary Fig. S2), which was consistent with autophagy activation during the onset of the confined cell death phase.

Autophagy-deficient mutants such as *atg5* are known to accumulate salicylic acid (SA) and display premature senescence and immune-related cell death (Yoshimoto et al. 2009). To determine whether the elevated colonization in these mutants is dependent on SA accumulation, we analyzed *NahG atg5* double mutants, which degrade SA via bacterial salicylate hydroxylase expression. *NahG atg5* plants retained the enhanced colonization phenotype observed in *atg5-3*, while *NahG* single mutants exhibited an intermediate response, more closely resembling wild type (Supplementary Fig. S3). These results indicate that increased susceptibility to *S. indica* in autophagy-deficient plants is largely independent of SA accumulation and signaling and instead reflects a direct role for autophagy in restricting fungal colonization.

### Autophagy promotes cell survival during dAdo-induced cell death

**Figure 2.**
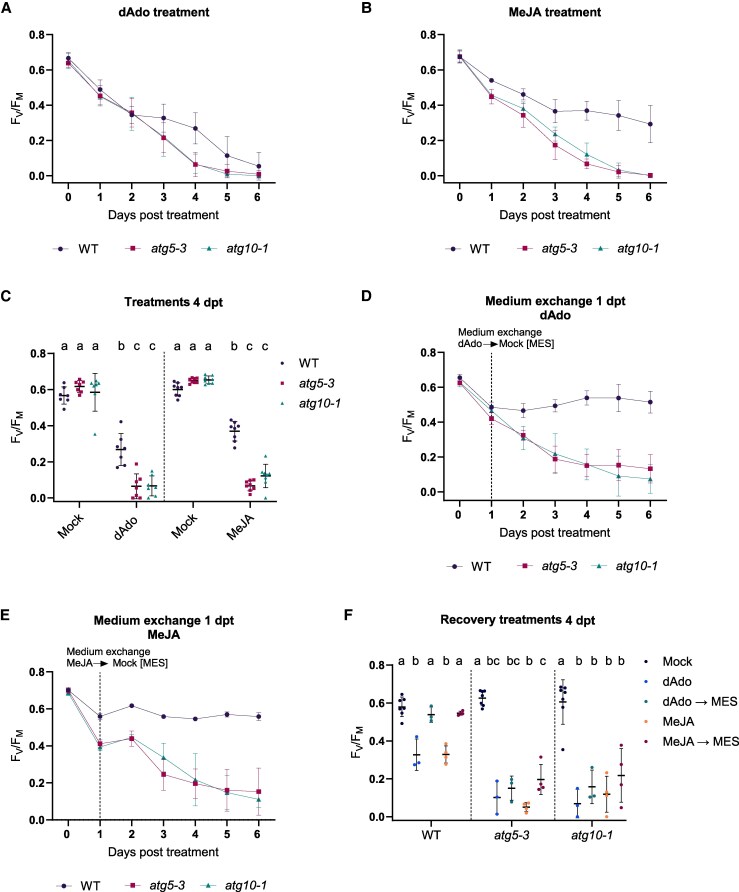
Autophagy promotes cell survival during dAdo-induced cell death. **A)** Photosystem II maximum quantum yield (*F*_V_/*F*_M_) of 9-d-old seedlings treated with mock (MES 2.5 mm buffer) or deoxyadenosine (dAdo, 500 *µ*m), measured by PAM fluorometry. Error bars represent ± Sd of the mean from 7 independent biological replicates. **B)**  *F*_V_/*F*_M_ of seedlings treated with mock or MeJA (500 *µ*m), serving as a positive control for cell death. Error bars represent ± Sd from 8 independent biological replicates. **C)** Quantification of *F*_V_/*F*_M_ at 4 d post treatment (dpt) in WT, *atg5-3*, and *atg10-1*. The plot (mean ± Sd) shows data from 7 to 8 biological replicates, each with 12 wells containing 3 seedlings per well. Different letters indicate statistically significant differences (*P* < 0.05; 1-way ANOVA with Tukey's Honestly Significant Difference (HSD) test). **D** and **E)**  *F*_V_/*F*_M_ measured after 24 h of deoxyadenosine (dAdo) or MeJA treatment followed by replacement with MES buffer, to assess recovery. Data represent means ± Sd from 3 (dAdo) and 4 (MeJA) biological replicates. **F)** Quantification of *F*_V_/*F*_M_ recovery at 4 d post-replacement in WT, *atg5-3*, and *atg10-1*. This extends the dAdo data shown in (C). The plot (mean ± Sd) represents 3 to 4 independent biological replicates (12 wells, 3 seedlings per well). Different letters denote significant differences (*P* < 0.05; 1-way ANOVA with Tukey's HSD test).

During the later stages of *S. indica* colonization, host root cells undergo confined cell death, a process modulated by the fungal-derived metabolite dAdo. This nucleoside is generated in the apoplast by the concerted action of the secreted effectors, *Si*E5NT and *Si*NucA, and imported into plant cells via the equilibrative nucleoside transporter ENT3, where it reprograms host immunity, cell fate, and metabolism (Dunken et al. 2024). To test whether autophagy contributes to host cell resilience during dAdo-induced stress, we monitored early physiological responses to dAdo exposure in autophagy-deficient mutants. We first assessed photosynthetic efficiency, measured as the ratio of variable to maximum fluorescence (*F*_V_/*F*_M_), as a proxy for plant health and cell viability in seedlings treated with dAdo. This metric is both reliable and quantifiable, reflecting systemic stress and serving as a general indicator of physiological sensitivity. Both *atg5-3* and *atg10-1* mutants exhibited significantly greater reductions in *F*_V_/*F*_M_ compared with WT, indicating increased sensitivity to dAdo in the absence of functional autophagy (Fig. 2, A to C; Supplementary Fig. S4). This hypersensitivity was further supported by reduced germination of *atg5-3* seeds upon dAdo exposure (Supplementary Fig. S5). Both mutants also showed increased sensitivity to methyl jasmonate (MeJA), a known inducer of senescence and regulated cell death (He et al. 2002), reinforcing a broader role for autophagy in suppressing stress-induced cell death (Fig. 2, B and C; Supplementary Fig. S4).

To determine whether this sensitivity extends to other components of the autophagy pathway, we examined *atg2-2* and *atg11-1*, which are impaired at distinct steps in autophagy initiation and vesicle formation. Both mutants also showed reduced germination or photosynthetic efficiency under dAdo treatment (Supplementary Figs. S5 and S6), indicating that impaired autophagy generally increases susceptibility to dAdo-induced stress. As previously shown for WT plants (Dunken et al. 2024), all genotypes exhibited a dose-dependent decline in *F*_V_/*F*_M_ with increasing dAdo concentrations (Supplementary Fig. S7), which is consistent with a gradual cellular stress response.

To assess whether autophagy supports recovery after transient stress, seedlings were treated with dAdo or MeJA for 24 h, then transferred to buffer, and *F*_V_/*F*_M_ was measured over time. WT plants fully recovered from both treatments, whereas *atg5-3* and *atg10-1* mutants failed to restore photosynthetic capacity (Fig. 2, D to F), indicating that autophagy is essential not only for mitigating initial damage but also to enable full recovery. Notably, prolonged dAdo exposure (48 h) abolished recovery even in WT plants, suggesting the existence of a critical threshold or “point-of-no-return” beyond which immunometabolic cell death becomes irreversible (Supplementary Fig. S8).

To determine whether the dAdo hypersensitivity of *atg5-3* is mediated by SA, we examined *NahG atg5* double mutants. These plants remained hypersensitive to dAdo, while *NahG* single mutants behaved like WT (Supplementary Fig. S9), confirming that the enhanced dAdo susceptibility of *atg5-3* is independent of SA accumulation.

Together, these results support a model in which autophagy promotes cellular tolerance to dAdo-induced stress. This is consistent with recent findings that dAdo activates an immunometabolic, ENT3-dependent cell death pathway in roots that bypasses classical immune-signaling mechanisms, such as ROS production and calcium influx (Dunken et al. 2024). In this context, autophagy likely functions to restore cellular homeostasis under immunometabolic stress, thereby preserving root cell viability and maintaining the integrity of the host–fungus interface during symbiosis.

### ENT3-dependent dAdo uptake drives symbiosis-mediated cell death in autophagy-deficient roots

**Figure 3.**
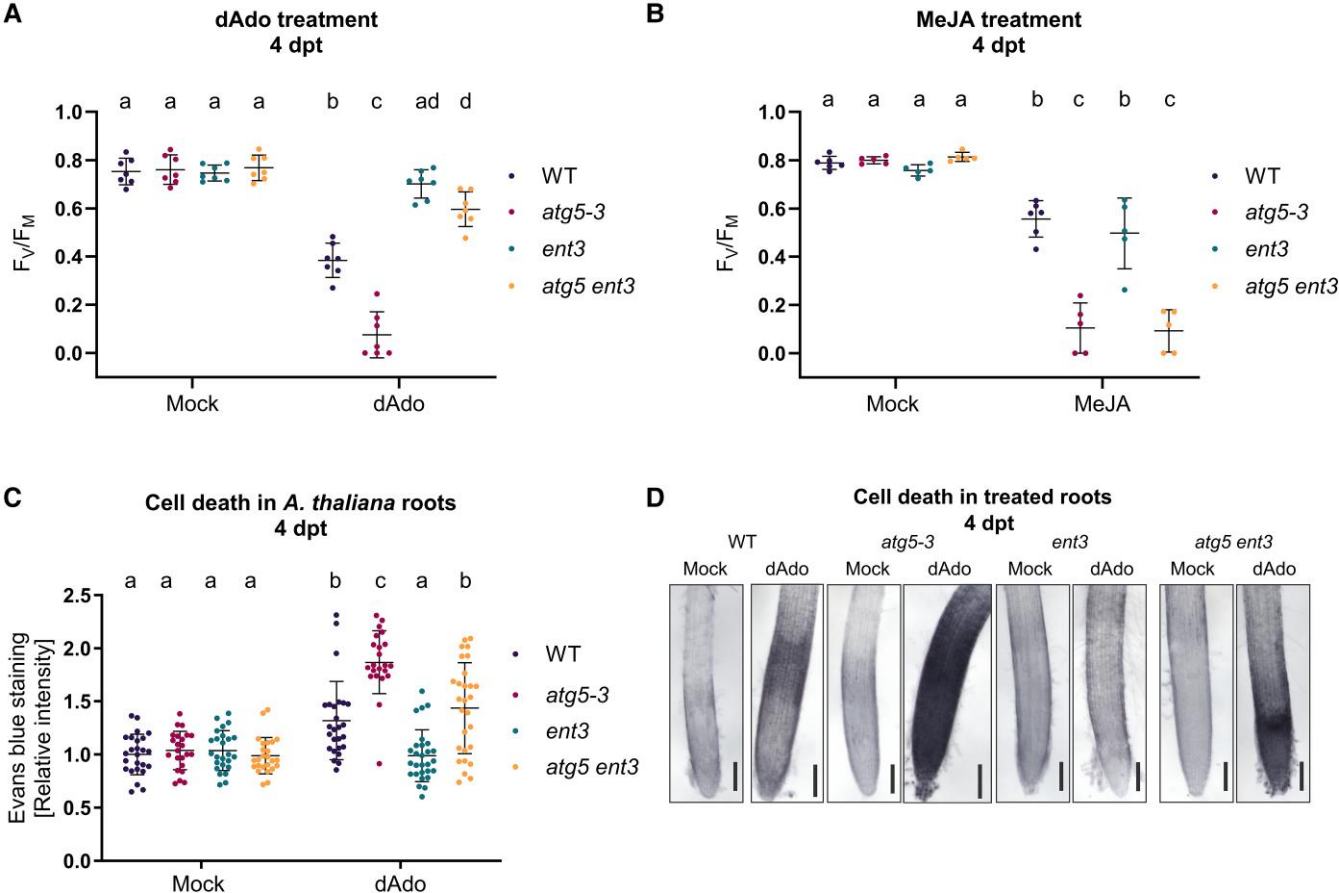
The double mutant *atg5 ent3* displays a dAdo resistance phenotype. **A)** Photosystem II maximum quantum yield (*F*_V_/*F*_M_) measured by PAM fluorometry in WT, *atg5-3*, *ent3*, and *atg5 ent3* seedlings at 4 dpt with mock (MES 2.5 mm buffer) or deoxyadenosine (dAdo, 500 *µ*m). The plot (mean ± Sd) represents data from 7 independent biological replicates, each consisting of 12 wells with 3 seedlings per well. Different letters indicate statistically significant differences (*P* < 0.05) determined by 1-way ANOVA with Tukey's HSD post hoc test. **B)**  *F*_V_/*F*_M_ values in the same genotypes as in (A), treated with mock or MeJA (500 *µ*m). The plot (mean ± Sd) represents data from 5 independent biological replicates. Different letters indicate statistically significant differences (*P* < 0.05) determined by 1-way ANOVA with Tukey's HSD post hoc test. **C)** Quantification of root tip cell death via Evans blue staining in 8-d-old seedlings treated with mock (milli-Q water) or deoxyadenosine (dAdo, 500 *µ*m) for 4 d. The plot (mean ± Sd) shows values relative to WT mock controls, based on 22 to 28 biological replicates. Different letters indicate statistically significant differences (*P* < 0.05) determined by Kruskal–Wallis test with post hoc Dunn test and Benjamini–Hochberg correction. **D)** Representative bright-field images of root tip cell death for the same genotypes and treatments as in (C). Scale bar: 500 *µ*m.

Previous work has shown that the uptake of dAdo via ENT3 is essential for initiating a non-canonical immunometabolic cell death pathway (Dunken et al. 2024). To test whether impaired dAdo import can alleviate the stress sensitivity of autophagy-deficient plants, we generated an *atg5-3 ent3* double mutant and examined its response to exogenous dAdo. Measurements of photosynthetic efficiency (*F*_V_/*F*_M_) revealed that *atg5-3 ent3* plants were significantly more tolerant to dAdo than *atg5-3*, closely resembling the resistance phenotype of *ent3* single mutants (Fig. 3A). In contrast, both *atg5-3* and the double mutant remained sensitive to MeJA, confirming that ENT3 specifically mediates dAdo- but not MeJA-induced cell death (Fig. 3B). Quantification of root cell death using Evans blue staining, which selectively permeates cells with ruptured plasma membranes (Vijayaraghavareddy et al. 2017), further supported this finding. While *atg5-3* roots showed extensive dAdo-induced cell death, both *ent3* and *atg5-3 ent3* roots displayed significantly reduced staining, with the double mutant exhibiting an intermediate phenotype (Fig. 3, C and D). These results indicate that dAdo uptake via ENT3 is a key driver of cell death in autophagy-deficient plants. The intermediate phenotype of *atg5-3 ent3* further suggests that minor alternative uptake routes, such as passive diffusion or entry through damaged membranes or low-affinity transporters, may contribute to residual dAdo accumulation and stress in the absence of autophagy. Consistently, residual adenosine uptake in the absence of ENT3 was previously shown in Traub et al. (2007).

**Figure 4.**
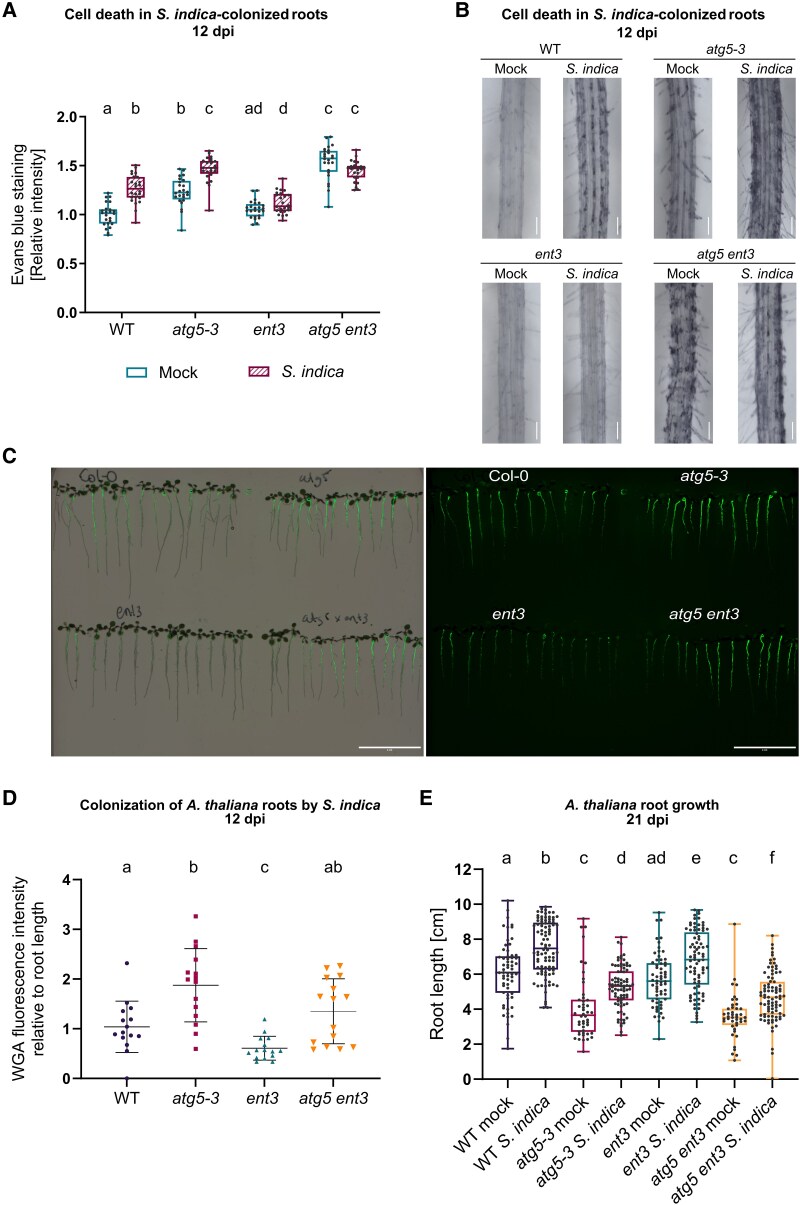
*S. indica*-mediated cell death is prevented in the double mutant *atg5 ent3*. **A)** Quantification of cell death in the root differentiation zone of WT, *atg5-3*, *ent3*, and *atg5 ent3* seedlings at 12 dpi with mock or *S. indica*, assessed by Evans blue staining. The box extends from the 25th to 75th percentiles, the center line is the median and the whiskers extend to the minimum and maximum values. Boxplots represent relative values normalized to WT mock (*n* = 6 biological replicates). **B)** Representative bright-field images of Evans blue-stained root differentiation zones for the same genotypes and treatments shown in (A). Scale bar: 500 *µ*m. **C)** Representative image of *A. thaliana* seedlings inoculated with *S. indica* via seed treatment and stained with WGA-AF488 to visualize fungal structures. Fluorescence was detected using the Odyssey M Imaging System. The left panel shows roots imaged in the brightfield channel; the right panel shows the corresponding image in the Alexa Fluor 488 channel. Scale bar: 4 cm. **D)** Quantification of extraradical *S. indica* colonization at 12 dpi on 1/10 PNM medium using WGA-AF488 fluorescence intensity. Values are normalized to root length and to WT mock controls. The plot (mean ± Sd) represents data from 15 biological replicates. **E)** Primary root length of WT, *atg5-3*, *ent3*, and *atg5 ent3* seedlings under mock or *S. indica* conditions at 21 dpi. Boxplots represent data from 43 to 92 biological replicates. The box extends from the 25th to 75th percentiles, the center line is the median and the whiskers extend to the minimum and maximum values. In all panels, different letters indicate statistically significant differences (*P* < 0.05), determined by Kruskal–Wallis test followed by Dunn's post hoc test with Benjamini–Hochberg correction.

To determine whether these effects extend to the colonization context, we assessed root cell viability at 12 dpi in plants inoculated with *S. indica*. As expected, both WT and *atg5-3* roots exhibited pronounced cell death in the differentiation zone upon colonization (Fig. 4, A and B). In contrast, *ent3* and *atg5-3 ent3* mutants showed Evans blue staining comparable to mock-treated controls. This pattern is consistent with dAdo-triggered, ENT3-dependent cell death during colonization and suggests that impaired dAdo uptake prevents symbiosis-induced cell death, regardless of autophagy status.

We next evaluated how these cellular phenotypes influence fungal colonization and the associated growth response under low-nutrient conditions (1/10 PNM), which promote mutualism and accentuate the effects of autophagy loss compared with nutrient-rich ½ MS medium (Lahrmann et al. 2013). Staining with the chitin-specific lectin WGA-AF488 at 12 dpi confirmed increased *S. indica* colonization in *atg5-3* roots relative to WT (Fig. 4, C and D). At 21 dpi, WT plants displayed significantly increased primary root length following colonization compared with mock controls. Strikingly, this growth promotion was also observed in both *atg5-3* and *atg5-3 ent3* plants (Fig. 4E), showing that autophagy is not required for the root growth phenotype conferred by *S. indica*. This supports a model where symbiosis modulates the classical growth–immunity trade-off in *Arabidopsis*.

**Figure 5.**
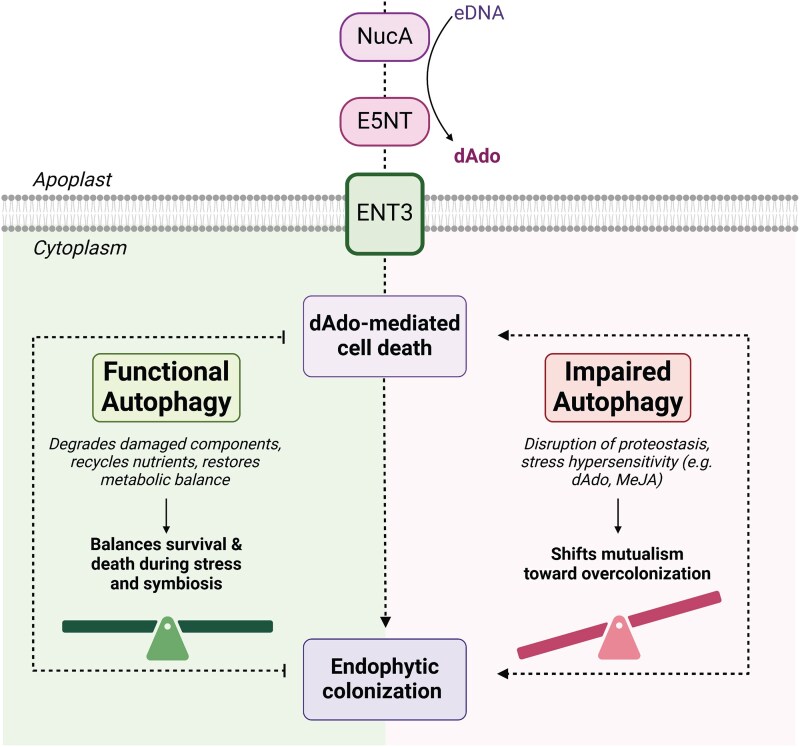
Model illustrating the crosstalk between autophagy and dAdo-induced cell death during *S. indica* colonization. Endophytic colonization of *A. thaliana* roots by the beneficial fungus *S. indica* involves a biotrophic phase followed by a restricted cell death-associated phase. Two secreted fungal enzymes, E5NT and NucA, generate the DNA-derived nucleoside deoxyadenosine (dAdo), which is imported into host cells via the equilibrative nucleoside transporter ENT3, triggering host signaling and a regulated cell death response. Autophagy, a key degradation and recycling pathway that maintains cellular homeostasis, is locally activated during this interaction. It limits fungal proliferation and mitigates dAdo-induced cell death. We propose that autophagy promotes host cell survival by clearing damaged components and protein aggregates, recycling nutrients, and supporting recovery from metabolic stress, thereby contributing to a balanced symbiotic relationship. Illustration created with BioRender.

Together, these findings indicate that autophagy and dAdo signaling intersect to regulate the balance between fungal accommodation and host cell survival. ENT3-mediated dAdo uptake promotes localized, regulated cell death, which facilitates fungal colonization, while autophagy mitigates the impact of this stress by promoting cell survival and preserving tissue integrity. The reduced colonization-associated cell death in *atg5-3 ent3* roots suggests that autophagy-deficient plants are not intrinsically prone to uncontrolled cell death but exhibit enhanced sensitivity to specific metabolic stress signals such as dAdo and MeJA. In this context, autophagy plays a critical role in maintaining cellular homeostasis during symbiosis, enabling the plant to accommodate a certain level of colonization-associated cell death and beneficial fungal proliferation while preserving root cell viability (Fig. 5).

## Discussion

Our study demonstrates that autophagy plays a central role in maintaining cellular homeostasis and regulating fungal accommodation during the interaction between *A. thaliana* and the beneficial root endophyte *S. indica*. Autophagy is activated in colonized root cells, particularly during the later stages of colonization associated with host cell death, where it acts to restrain fungal overproliferation and prevents extensive tissue damage. Microscopy and immunoblotting confirmed local induction of autophagy at sites of fungal contact. The detection of colonization-specific mCherry-ATG8E cleavage products even in *atg5-1* backgrounds suggests ATG5-independent degradation pathways, potentially involving host- or fungus-derived proteases active during the regulated cell death phase. These findings support a model in which autophagy is engaged at the host–microbe interface to promote cellular stability during symbiosis.

Using autophagy-deficient mutants, we show that loss of ATG5 or ATG10 leads to increased fungal colonization and enhanced susceptibility to immunometabolic cell death, particularly at the onset of the cell death-associated phase. The upregulation of immune- and metabolism-related stress genes, including *WRKY33*, *CYP81F2*, and *AT1G58420*, in these mutants likely reflects amplified immune signaling triggered by elevated fungal burden. This suggests that autophagy not only restricts fungal overgrowth but may also modulate host immunometabolic signaling cascades during root symbiosis.

We further establish a functional connection between autophagy and dAdo-induced cell death, a process triggered by fungal apoplastic effectors and dependent on the host nucleoside transporter ENT3 (Dunken et al. 2024). Autophagy-deficient mutants (*atg5-3*, *atg10-1*, *atg2-2*, and *atg11-1*) display increased sensitivity to dAdo and fail to recover following transient exposure, indicating impaired resilience under metabolic stress. Importantly, dAdo-induced cell death is attenuated in *atg5-3 ent3* double mutants, confirming that ENT3-mediated dAdo uptake is required to activate this pathway. Although ENT3 appears to be the primary route for dAdo uptake, residual sensitivity in the *atg5-3 ent3* double mutant suggests the possibility of passive diffusion or uptake by other nucleoside transporters like ENT6 (Wormit et al. 2004). Similar residual uptake of adenosine has previously been reported for the *ent3* mutant (Traub et al. 2007). While our results do not rule out alternative uptake routes, their contribution seems minor compared with ENT3. The pronounced sensitivity of the root tip to dAdo, which we leveraged for early stress quantification, aligns with findings from animal systems where dAdo-triggered cell death preferentially affects young, actively dividing cells (Carson et al. 1979; Thammavongsa et al. 2013). We also observed that *S. indica* is frequently present in the rhizosphere near the root tip, a region enriched in eDNA. In this context, the fungus may release diffusible signals such as dAdo that prime host cells release for regulated cell death, ultimately facilitating intracellular fungal accommodation in the more differentiated cells of the maturation zone. Although autophagy-deficient plants accumulate SA, our data show that dAdo hypersensitivity and elevated colonization are SA-independent, as *NahG atg5* plants retain these phenotypes. These autophagy-deficient mutants also display enhanced sensitivity to MeJA, a known inducer of senescence and cell death, further supporting autophagy's established role in suppressing stress-amplifying signals and promoting survival (Liu et al. 2009; Ma et al. 2021; Thirumalaikumar et al. 2021).

Interestingly, *atg5-3 ent3* mutants exhibit diminished dAdo-triggered cell death, yet fungal colonization remains elevated, showing an intermediate phenotype between *atg5-3* and WT (Fig. 4D). This may be explained by the elevated baseline cell death observed in *atg5-3*, and even more prominently in *atg5-3 ent3*, under mock conditions, suggesting that autophagy may also regulate developmental or homeostatic cell death in root tissues. The increased basal cell death observed in *atg5-3 ent3* roots compared with *atg5-3*, in the absence of fungal colonization (Fig. 4A), points to a functional connection between autophagy and purinergic metabolism. Given ENT3's role in nucleotide salvage pathways (Traub et al. 2007; Cornelius et al. 2012), its loss in an autophagy-deficient background may exacerbate purine imbalance, thereby intensifying host metabolic stress and contributing to both cell death and enhanced fungal colonization. This interpretation aligns with previous findings that cellular purine levels influence autophagy and stress signaling via target of rapamycin (TOR)-related pathways (Hoxhaj et al. 2017; Kazibwe et al. 2020). In this context, *S. indica* may encounter a physiologically permissive environment in *atg5-3 ent3* roots that reduces its reliance on dAdo-mediated cell death to establish colonization, potentially explaining the reduced Evans blue staining observed during symbiosis. Thus, the apparent suppression of colonization-associated cell death, despite high fungal colonization, in the double mutant likely reflects altered host–microbe dynamics in a root system already primed for cell death due to underlying metabolic imbalance. This concept is well-established in animal systems, where disruptions in purinergic metabolism have been shown to modulate autophagy and influence susceptibility to microbial infections (Tattoli et al. 2012; Manzanillo et al. 2013). Autophagy plays a critical role in host defense against intracellular pathogens, and its impairment leads to increased pathogen survival and replication (Gutierrez et al. 2004 ; Deretic et al. 2013). The crosstalk between autophagy and cellular metabolism, including purine turnover, is essential for maintaining immune homeostasis (Rabinowitz and White 2010; Guo et al. 2016). Moreover, mutations in autophagy-related genes have been linked to enhanced vulnerability to infection and inflammatory disease (Levine et al. 2011; Cadwell 2016).

Despite enhanced fungal colonization and increased host stress susceptibility, autophagy-deficient plants retain the root growth-promoting effects of *S. indica* under nutrient-limited conditions. This retention, despite heightened stress and cell death, indicates that the underlying growth benefits are largely independent of autophagy-mediated stress resilience. Instead, these benefits may arise from fungal modulation of host developmental programs. Previous studies have shown that Sebacinales fungi can alter phytohormone signaling such as auxin and cytokinin (Vadassery et al. 2008; Xu et al. 2018; Zhang et al. 2018) and enhance nutrient acquisition (Rong et al. 2023), both of which directly affect plant developmental pathways. Such mechanisms could operate in parallel to stress responses, thereby sustaining root growth promotion even when cellular homeostasis is compromised.

The decoupling of mutualistic benefits from colonization control highlights distinct regulatory layers governing symbiotic outcomes. Together, our findings show that autophagy restricts fungal proliferation and mitigates dAdo-induced cell death without compromising beneficial traits of the interaction. The increased stress sensitivity and baseline cell death observed in autophagy mutants underscore its broader role in maintaining cellular homeostasis, particularly under fluctuating environmental or microbial conditions.

In summary, autophagy acts as a critical determinant of root cell viability during symbiosis. It limits dAdo-induced, ENT3-dependent cell death and contains fungal proliferation. By integrating immune and metabolic stress responses, autophagy enables the plant to accommodate beneficial colonization while preserving tissue integrity (Fig. 5). These findings reveal how a core cellular process shapes the outcome of root–microbe interactions and underscore the importance of stress resilience and purinergic metabolism in maintaining mutualistic balance.

## Materials and methods

### Plant lines

Arabidopsis (*A. thaliana*) ecotype Columbia (Col-0) was used as a WT control. The T-DNA insertion mutant lines in this study are: *atg5-1* (AT5G17290) SAIL-129B07, *atg5-3* SALK-020601C (Thompson et al. 2005; Have et al. 2019), *atg10-1* (AT3G07525) SALK-084434, *atg11-1* (AT4G30790) SAIL-1166G10, and *ent3* (AT4G05120) SALK-204257C. *atg2-2* (AT3G19190) is an EMS-mutant in a Col-0 background (Wang et al. 2011). The transgenic lines *pUbi::mCherry-ATG8E* in Col-0 WT and *atg5-1* background (Stephani et al. 2020), *NahG* and *NahG atg5* transgenic lines were provided by Dr. Yasin Dagdas lab. The double mutant *atg5 ent3* was obtained by crossing *atg5-3* and *ent3* lines. All transgenic lines were genotyped by PCR and homozygous lines were isolated.

### Plant-growth conditions

Surface-sterilized seeds of *A. thaliana* were germinated and grown on ½ MS (Murashige-Skoog Medium, with vitamins, pH 5.7) containing 0.5% (w/v) sucrose and 0.4% (w/v) Gelrite (Duchefa, Haarlem, the Netherlands) and stratified in the dark for 3 d at 4 °C. Plants were grown under short day conditions (8 h light, 16 h dark) with 130 *µ*mol m^−2^ s^−1^ of light and 22 °C/18 °C.

### Fungal strains and culturing techniques

*S. indica* strain DSM11827 (German Collection of Microorganisms and Cell Cultures, Braunschweig, Germany) was grown on a complete medium (CM) containing 2% (w/v) glucose and 1.5% (w/v) agar at 28 °C as described (Hilbert et al. 2012). For confocal microscopy studies, the *S. indica* strain constitutively expressing a *S. indica* codon-optimized GFP gene was used (Hilbert et al. 2012).

### Fungal inoculation

For seedling inoculation, 8-d-old *A. thaliana* seedlings were transferred to ½ MS and 0.4% (w/v) Gelrite plates. Nine-day-old seedlings, specifically the roots and the surrounding area, were inoculated with 800 *µ*L containing 5 × 10^5^  *S. indica* chlamydospores/mL. Control plants were inoculated with sterile milli-Q water as mock treatment. At the indicated time points, a 4 cm root section was harvested starting 0.5 cm below the shoot. Colonized roots were washed thoroughly to remove extraradical hyphae and frozen in liquid nitrogen. Each biological replicate contains 3 plates with 15 seedlings each. For seed inoculation, surface-sterilized *A. thaliana* seeds were incubated in 1 mL containing 5 × 10^5^  *S. indica* chlamydospores/mL or sterile milli-Q water for 1 h followed by their distribution on ½ MS and 0.4% (w/v) Gelrite plates.

### RNA extraction and real-time quantitative PCR analysis

Total RNA was extracted from colonized or mock-treated ground plant root material using TRIzol reagent (Invitrogen, Thermo Fisher Scientific, Schwerte, Germany) according to the manufacturer's instructions and digested with DNase I to prevent genomic DNA contamination (Thermo Fisher Scientific). cDNA was synthesized with 1 *µ*g total RNA primed with Oligo-dT and random hexamers primers using the First Strand cDNA Synthesis Kit (Thermo Fisher Scientific). Quantitative real-time PCR was performed using the 2× GoTaq qPCR master mix (Promega, Walldorf, Germany) with 10 ng of cDNA template and 0.5 *µ*m of each oligonucleotide in a final volume of 15 *µ*L. Reactions were amplified in a CFX connect real-time system (BioRad, Munich, Germany) according to the following protocol 95 *°*C 3 min, 95 *°*C 15 s, 59 *°*C 20 s, 72 *°*C 30 s, 40 cycles and a melting curve analysis. Relative expression was calculated using the 2^−Δ*Ct*^ method. Sequences of all primers can be found in Supplementary Table S1.

### PAM fluorometric measurements

For chlorophyll fluorescence analysis, 8-d-old *A. thaliana* seedlings were transferred to 24-well plates (3 per well) containing 2 mL of 2.5 mm MES buffer (pH 5.6). After overnight recovery, seedlings were treated with mock (MES 2.5 mm buffer), dAdo, or MeJA (Sigma-Aldrich, Taufkirchen, Germany) to a final concentration of 500 *µ*m. Maximum quantum yield of photosystem-II (*F*_V_/*F*_M_) of dark-adapted samples was quantified using pulse amplitude modulation (PAM) fluorometry (M-Series PAM fluorometer, Heinz Walz GmbH, Effeltrich, Germany). Data were analyzed using ImagingWin software (V2.56p; Walz, Germany). All measurements were performed for each genotype and across all treatments to ensure consistent comparison and minimize bias.

### Seed germination assays

Surface-sterilized seeds of *A. thaliana* were transferred to 24-well plates, containing 2 mL of 1/10 PNM (Plant Nutrition Medium, pH 5.6). The medium was supplemented with 500 *µ*m dAdo or mock (2.5 mm MES, pH 5.6). Ten seeds were placed into each well, and after 2 d of stratification, they were grown under short day conditions. The growth of the seedlings was monitored via PAM fluorometry at 7, 14, and 21 d after transfer to the growth chamber. The photosynthetic active area was analyzed using the software Fiji (ImageJ) (Schindelin et al. 2012). All measurements were performed for each genotype and across all treatments to ensure consistent comparison and minimize bias.

### Root length measurements

Surface-sterilized *A. thaliana* seeds were inoculated with mock or *S. indica* spores on plates containing 1/10 PNM as described in Lahrmann et al. (2013). Twenty-one days after inoculation, scans of the square plates containing seedlings were taken. Images were analyzed using Fiji (ImageJ) to measure the length of the primary root (centimeter) of seedlings developing true leaves. All physiological measurements were performed for each genotype and across all treatments to ensure consistent comparison and minimize bias.

### Extraradical colonization assays

Quantification of extraradical colonization of *S. indica* on *A. thaliana* was performed on seed-inoculated plants grown for 12 d and stained with the chitin-binding lectin stain Wheat Germ Agglutinin conjugated with Alexa Fluor 488 (WGA-AF 488, Invitrogen Thermo Fisher Scientific). Mock and *S. indica*-treated seedlings were stained directly on a plate with 1× phosphate-buffered saline (PBS) solution containing WGA-AF 488 (5 *µ*L/mL from 1 mg/mL stock solution) and incubated for 1 to 2 min. Subsequently, the roots were washed with 1× PBS solution. The stained seedlings were transferred to a new fresh plate, and fluorescence detection was conducted using an Odyssey M Imaging System (LI-COR Biosciences). WGA-AF 488 fluorescence intensity was quantified using ImageJ by subtracting background signal and normalizing to the root length of the different genotypes. All colonization measurements were performed for each genotype and across all treatments to ensure consistent comparison and minimize bias.

### Cell death staining with Evans blue

Root cell death was quantified using Evans blue dye (Sigma-Aldrich) to assess *S. indica*- or dAdo-induced cell death at the indicated time points. Treated roots were washed 3 times with milli-Q water before staining with 2.5 mm Evans blue solution in 0.1 m CaCl_2_ pH 5.6 for 15 min, based on a modified version of the protocol described in Vijayaraghavareddy et al. (2017). After 1 h of washing, root images were taken using a Leica M165 FC stereo microscope. The microscopy of *S. indica*-colonized samples was performed using the differentiation zone of the *A. thaliana* roots, and the microscopy of the roots treated with dAdo corresponds to the root tip. Quantification of cell death was performed using Fiji (ImageJ). Microscopy and quantifications were performed for each genotype and across all treatments to ensure consistent comparison and minimize bias.

### Autophagic flux assay

For carbon starvation treatment, *A. thaliana* seedlings expressing *pUbi::mCherry-ATG8E* in WT or in the *atg5-1* KO mutant background were grown on ½ MS containing 1% (w/v) sucrose and 0.4% (w/v) Gelrite. After 7 d, seedlings were transferred to ½ MS without sucrose and plates were covered with an aluminum foil and grown under the same conditions for 9 d (Stephani et al. 2020). Whole seedlings were harvested and frozen in liquid nitrogen. For analysis during *S. indica* colonization, surface-sterilized *A. thaliana* seeds were inoculated with mock or *S. indica* treatment. After 10 d, root tissue was harvested and frozen in liquid nitrogen.

### Protein extraction and immunoblot analysis

Frozen samples were homogenized with a bead mill (TissueLyser II, Qiagen) for 2 min (frequency 30 hertz). 1× lysis buffer (2×) lysis buffer: 300 mm NaCl, 100 mm HEPES pH 7.4, 2 mm EDTA, 2% Triton X-100) with 1× plant protease inhibitor cocktail (Sigma-Aldrich) and 1 mm phenylmethylsulfonyl fluoride was added, and samples were vortexed. The lysates were cleared by centrifugation at 13,000 × *g* for 10 min at 4 °C. Protein concentration was determined using the Pierce BCA Protein Assay Kit (Thermo Fisher Scientific). The protein extract was mixed with Laemmli buffer (6×) containing beta-mercaptoethanol and then boiled for 10 min at 95 °C. SDS-PAGE was performed with 10% gels and 1× Tris Glycine-SDS buffer (BioRad, Munich, Germany). Semi-dry blotting was performed on PVDF membranes. Membranes were blocked with 3% BSA (VWR, Darmstadt, Germany) in TBS and 0.05% Tween 20% (v/v) (TBS-T) for 1 h at room temperature. After incubation overnight at 4 °C with the primary antibody anti-mCherry (1:1,000, 5993, BioVision) diluted in 1× PBS, the membranes were washed 3 times with TBS-T. After a 40 min incubation with the secondary antibodies IRDye 680RD/800CW (1:10,000, LI-COR) diluted in 3% BSA TBS-T, the membranes were washed 3 times with TBS-T and finally rinsed with 1× PBS. Fluorescent western blot detection was performed using an OdysseyDLx (LI-COR Biosciences GmbH, Bad Homburg vor der Höhe, Germany).

### Confocal imaging and image quantification

A TCS SP8 confocal microscope (Leica, Wetzlar, Germany) was used for confocal laser scanning microscopy on living cells. mCherry was excited by a laser at 561 nm and the emitted light was detected with a hybrid detector (HyD2) at 602 to 638 nm. The mCherry-ATG8-labelled punctate structures were counted in a 63× captured image size (184.52 *µ*m × 184.52 *µ*m) considering maximal projections of 8 to 10 frames with 4 *µ*m step size. GFP was excited by a laser at 488 nm, and the emitted light was detected with a hybrid detector (HyD1) at 513 to 527 nm.

### Statistical analysis

Analysis was performed using GraphPad Prism software (v.9.4.1 for Windows) or RStudio (R v.4.1.1 for Windows). The detailed statistical method is given in the figure legends.

### Transcriptomic analysis (RNA sequencing)

*A. thaliana* Col-0 WT roots were inoculated with either mock (milli-Q water) or *S. indica*, and harvested at 1, 3, 6, and 10 dpi. Each condition was represented by 3 biological replicates. Stranded RNA-Seq libraries were prepared and quantified via qPCR, and sequencing was performed at the U.S. Department of Energy Joint Genome Institute (JGI) under project proposal ID: 505829 (Eichfeld et al. 2024). Raw reads were processed through the JGI quality control pipeline, including filtering and trimming. Filtered reads were aligned to the *A. thaliana* TAIR10 reference genome using HISAT2 (Kim et al. 2015), and gene-level counts were obtained with featureCounts (Liao et al. 2014). Differential gene expression analysis was carried out using DESeq2 (Love et al. 2014). Autophagy-associated genes were identified based on gene ontology annotations (Denny et al. 2018) and retrieved from the TAIR10 annotation release dated May 1 2023. The data can be found in Supplementary Table S2.

### Accession numbers

Sequence data from this article can be found in the GenBank/EMBL data libraries under accession numbers AT4G05120 (AtENT3), AT3G19190 (AtATG2), AT5G17290 (AtATG5), AT2G45170 (AtATG8E), AT3G07525 (AtATG10), and AT4G30790 (AtATG11).

## Supplementary Material

kiaf590_Supplementary_Data

## Data Availability

RNA-seq data shown in this study have been deposited in the Gene Expression Omnibus (GEO) database under the accession numbers GEO:GSE209761 and GEO:GSM6394981. Microscopy data reported in this paper will be shared by the lead contact upon request.This paper does not report original code. Any additional information required to reanalyze the data reported in this paper is available from the lead contact upon request.

## Supplementary data

The following materials are available in the online version of this article.

**Supplementary Figure S1.** Expression of immune marker genes in *A. thaliana* WT and autophagy mutants *atg5-3* and *atg10-1*.

**Supplementary Figure S2.** Expression of autophagy-associated genes during *S. indica* colonization.

**Supplementary Figure S3.** Colonization by *S. indica* at 12 dpi in *A. thaliana* WT, *NahG* and *NahG atg5* mutants.

**Supplementary Figure S4.** Autophagy mutants *atg5-3* and *atg10-1* show increased sensitivity to dAdo treatment.

**Supplementary Figure S5.** Seed germination assay in *A. thaliana* WT and autophagy mutants *atg5-3* and *atg2-2*.

**Supplementary Figure S6.** Autophagy mutant *atg11-1* show increased sensitivity upon dAdo treatment.

**Supplementary Figure S7.** dAdo dose-dependent response in autophagy mutants.

**Supplementary Figure S8.** dAdo recovery assay in *A. thaliana* WT and autophagy mutants *atg5-3* and *atg10-1*.

**Supplementary Figure S9.** Increased sensitivity to dAdo treatment in *atg5* mutants is independent of SA accumulation.

**Supplementary Table S1.** Primers used in real-time qPCR analysis.

**Supplementary Table S2.** Result of differential expression analysis of Arabidopsis autophagy-associated genes during *S. indica* colonization.

## Dive Curated Terms

The following phenotypic, genotypic, and functional terms are of significance to the work described in this paper:

ATG10 Gramene: AT3G07525
ATG10 Araport: AT3G07525

AT1G58420 Gramene: AT1G58420
AT1G58420 Araport: AT1G58420

CYP81F2 Gramene: AT5G57220
CYP81F2 Araport: AT5G57220

atg2 Gramene: AT3G51800
atg2 Araport: AT3G51800

MES CHEBI: CHEBI:39010

WRKY33 Gramene: AT2G38470
WRKY33 Araport: AT2G38470

ENT3 Gramene: AT4G05120
ENT3 Araport: AT4G05120

GFP Gramene: green fluorescent protein
GFP Araport: green fluorescent protein

root AmiGo: PO:0009005

CLSM Gramene: Confocal Laser-Scanning Microscopy
CLSM Araport: Confocal Laser-Scanning Microscopy
