# Supplementary material for: Autophagy restricts symbiosis-associated cell death and regulates colonization by Serendipita indica in Arabidopsis
Source: Plant Physiol. 2025 Nov 18;199(4):kiaf590. doi: 10.1093/plphys/kiaf590 (PMC12671557; doi:10.1093/plphys/kiaf590)
Supplement: kiaf590_Supplementary_Data [file kiaf590_supplementary_data.zip › Supplementary Figures_PlantPhys_PZ.docx]

**Supplementary figures**


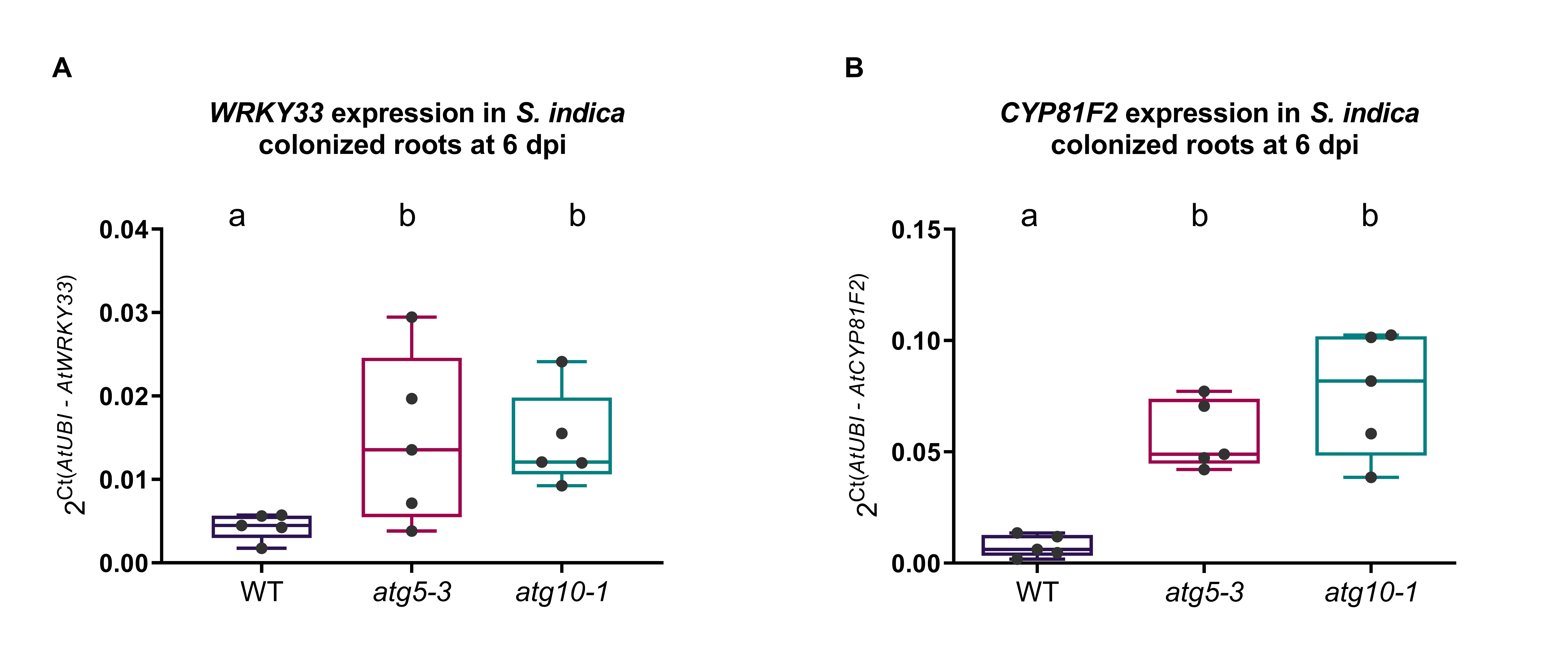


**Supplementary Figure S1: Expression of immune marker genes in *A. thaliana* WT and autophagy mutants *atg5-3* and *atg10-1*.**

(**A-B**) Expression of *WRKY33* and *CYP81F2* marker genes in WT (wild type) and autophagy mutants *atg5-3* and *atg10-1* in *Serendipita indica*-colonized roots at 6 dpi (days post inoculation). Expression was normalized to the reference gene (*AtUBI*) using cDNA as template and the 2^−∆Ct^ method. The box extends from the 25th to 75th percentiles, the center line is the median and the whiskers extend to the minimum and maximum values. The values represent data from 5 independent biological replicates. Different letters indicate statistically significant differences (p < 0.05; Kruskal-Wallis test followed by Dunn's post-hoc test with Benjamini-Hochberg correction).





**Supplementary Figure S2: Expression of autophagy-associated genes during *S. indica* colonization.**

Heatmap showing transcript abundance of autophagy-associated genes in *A. thaliana* WT (wild type) roots at 1, 3, 6, and 10 dpi (days post inoculation) of *Serendipita indica* (*S.i.*) or mock treatment. Genes with an average expression >1 TPM (transcripts per million) across all samples were included. Expression values represent the z-score of log₂-transformed (TPM + 1) values. For each condition, the average expression across three biological replicates is shown. Differential expression was analyzed using DESeq2 (pairwise comparisons of *S.i.-*treated vs. mock-treated samples at each timepoint). See Supplementary Table S2 for full results. (Zuccaro & Langen, 2020; Eichfeld et al., 2024).


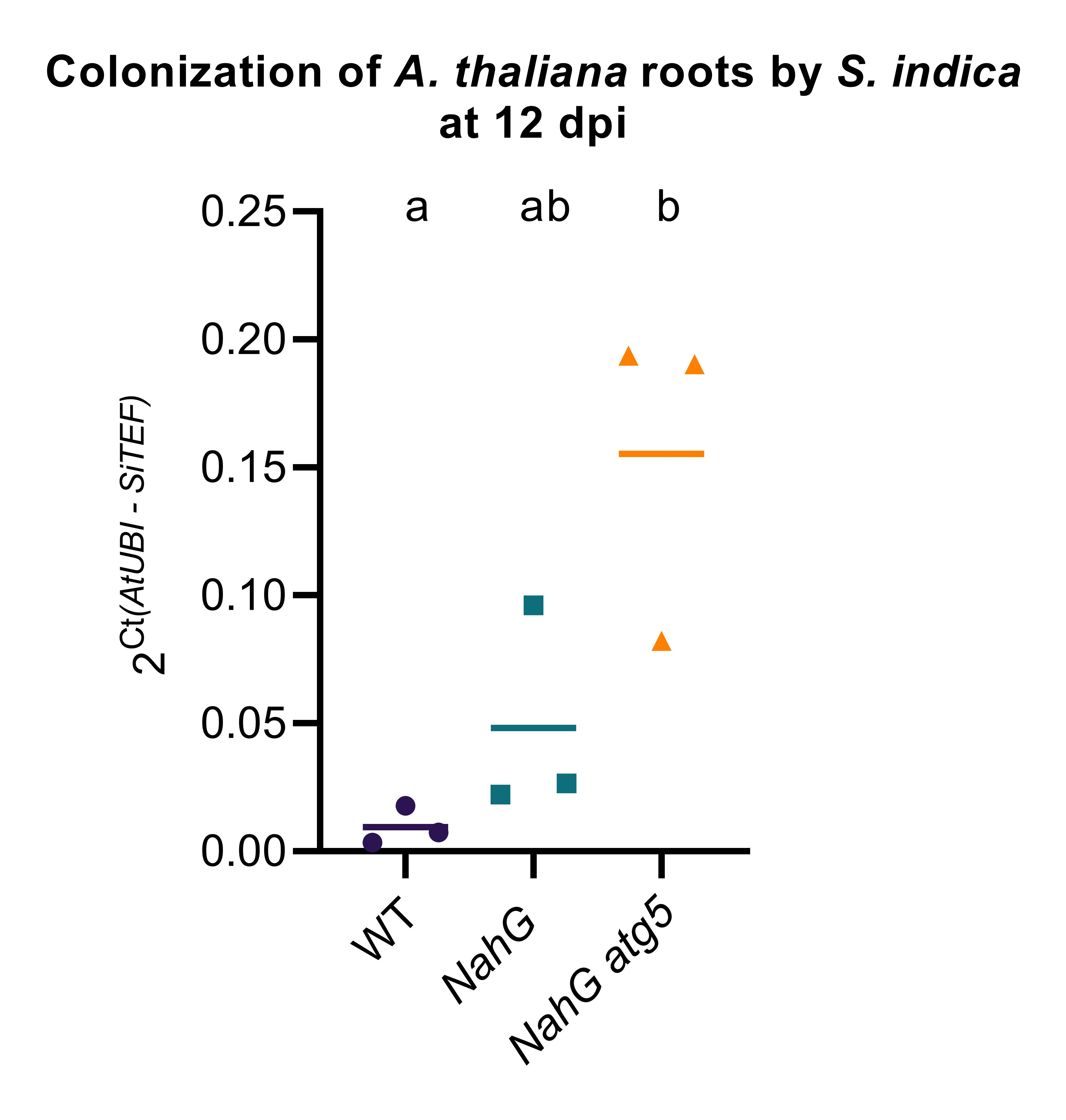


**Supplementary Figure S3: Colonization by *S. indica* at 12 dpi in *A. thaliana* WT, *NahG* and *NahG atg5* mutants.**

Quantification of endophytic colonization in roots of *Serendipita indica* seed-inoculated *Arabidopsis thaliana* plants at 12 dpi (days post inoculation) using RT-qPCR. Fungal biomass was calculated as the ratio of *S. indica* (*SiTEF*) to host (*AtUBI*) transcript levels using cDNA as template and the 2^−ΔCt^ method. The plot (mean) represents data from 3 independent biological replicates. Different letters indicate statistically significant differences (p < 0.05; Kruskal-Wallis test with Dunn’s post-hoc test and Benjamini-Hochberg correction).


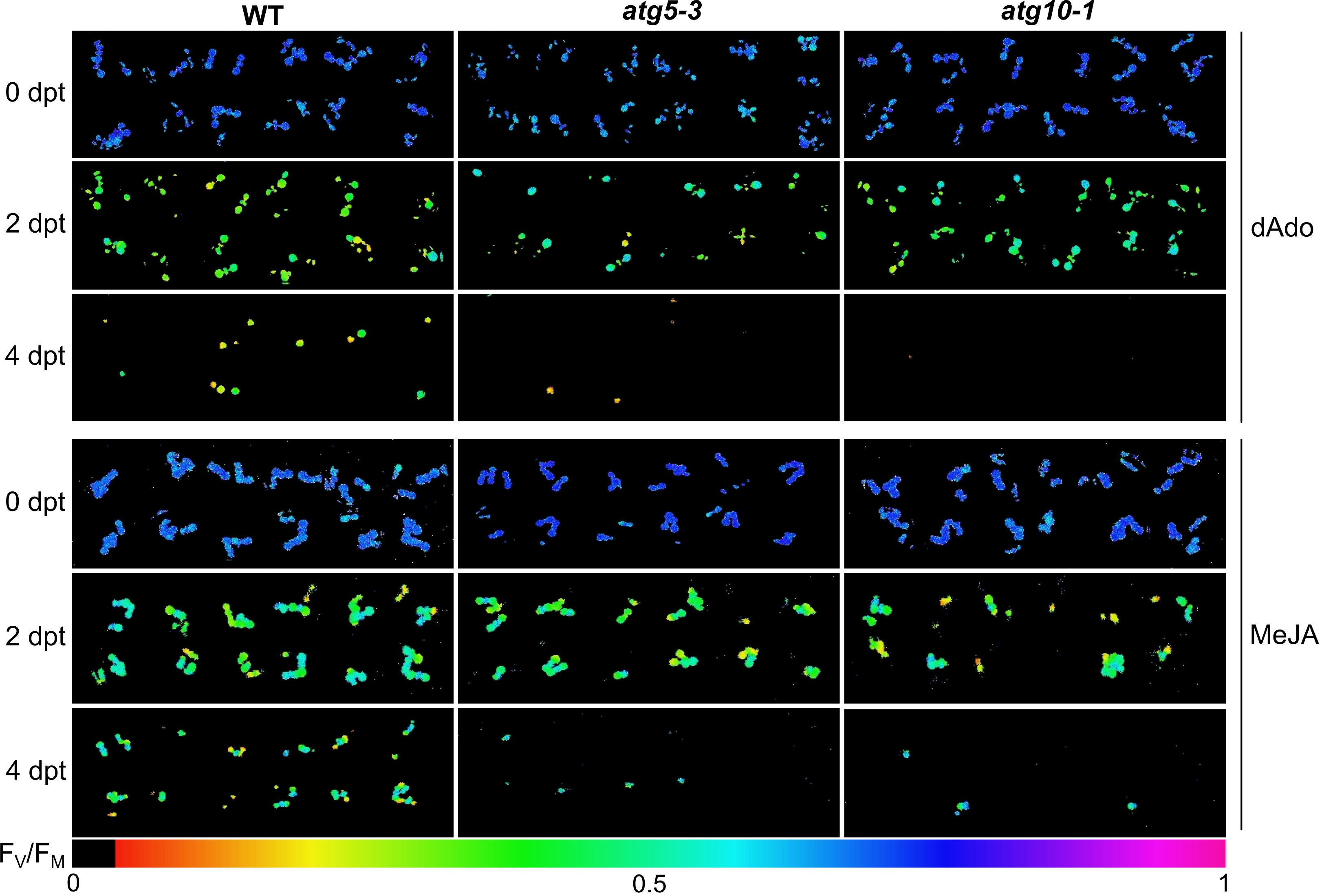


**Supplementary Figure S4: Autophagy mutants *atg5-3* and *atg10-1* show increased sensitivity to dAdo treatment.**

Visualization of photosystem II maximum quantum yield (F_V_/F_M_) in WT (wild type) and autophagy mutants *atg5-3* and *atg10-1* following treatment with deoxyadenosine (dAdo) or methyl jasmonate (MeJA), both at 500 µM, at 0, 2, and 4 dpt (days post treatment). F_V_/F_M_ values are represented using a color scale (shown below). Each treatment consisted of 12 wells, with three seedlings per well.


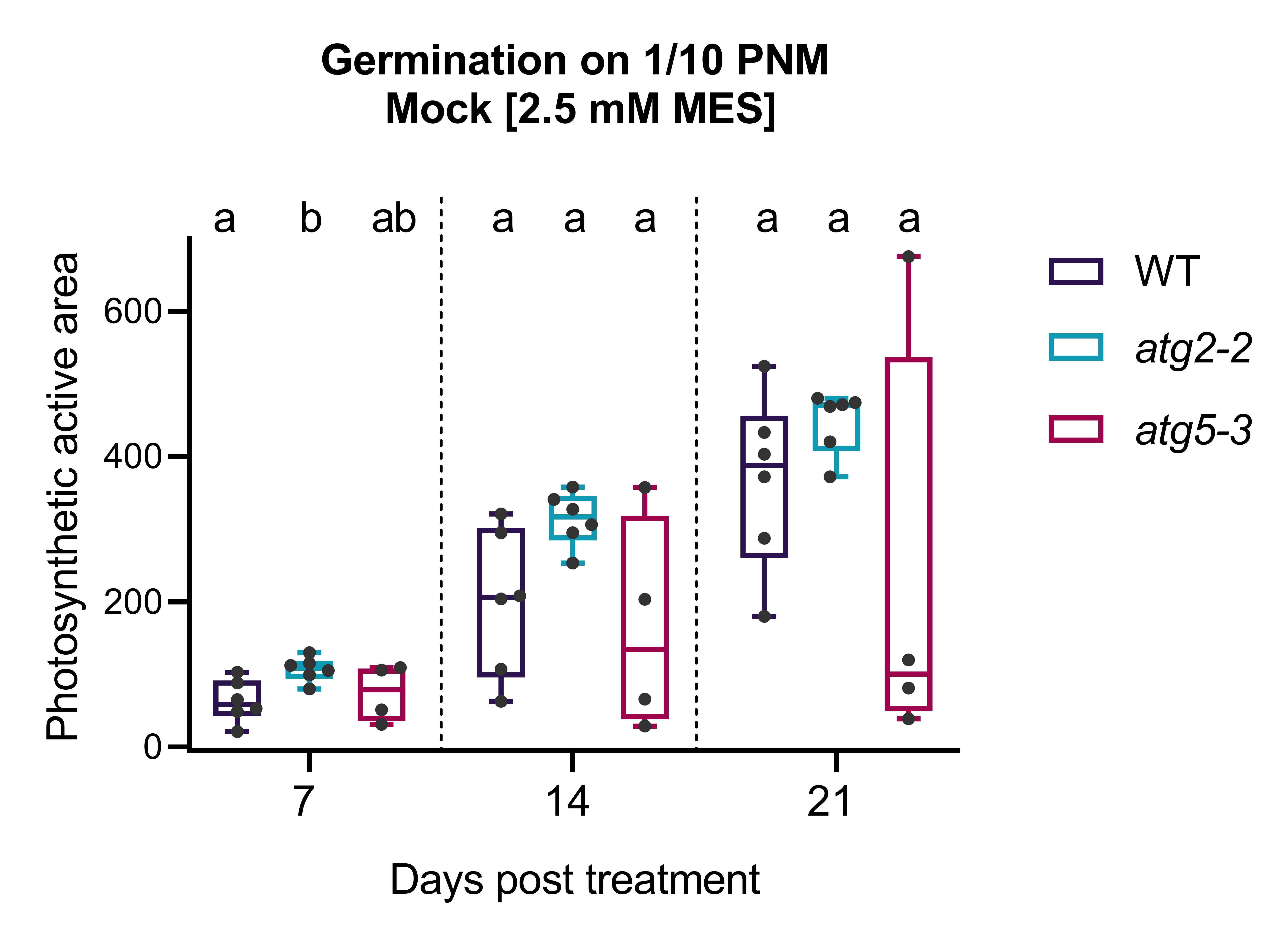

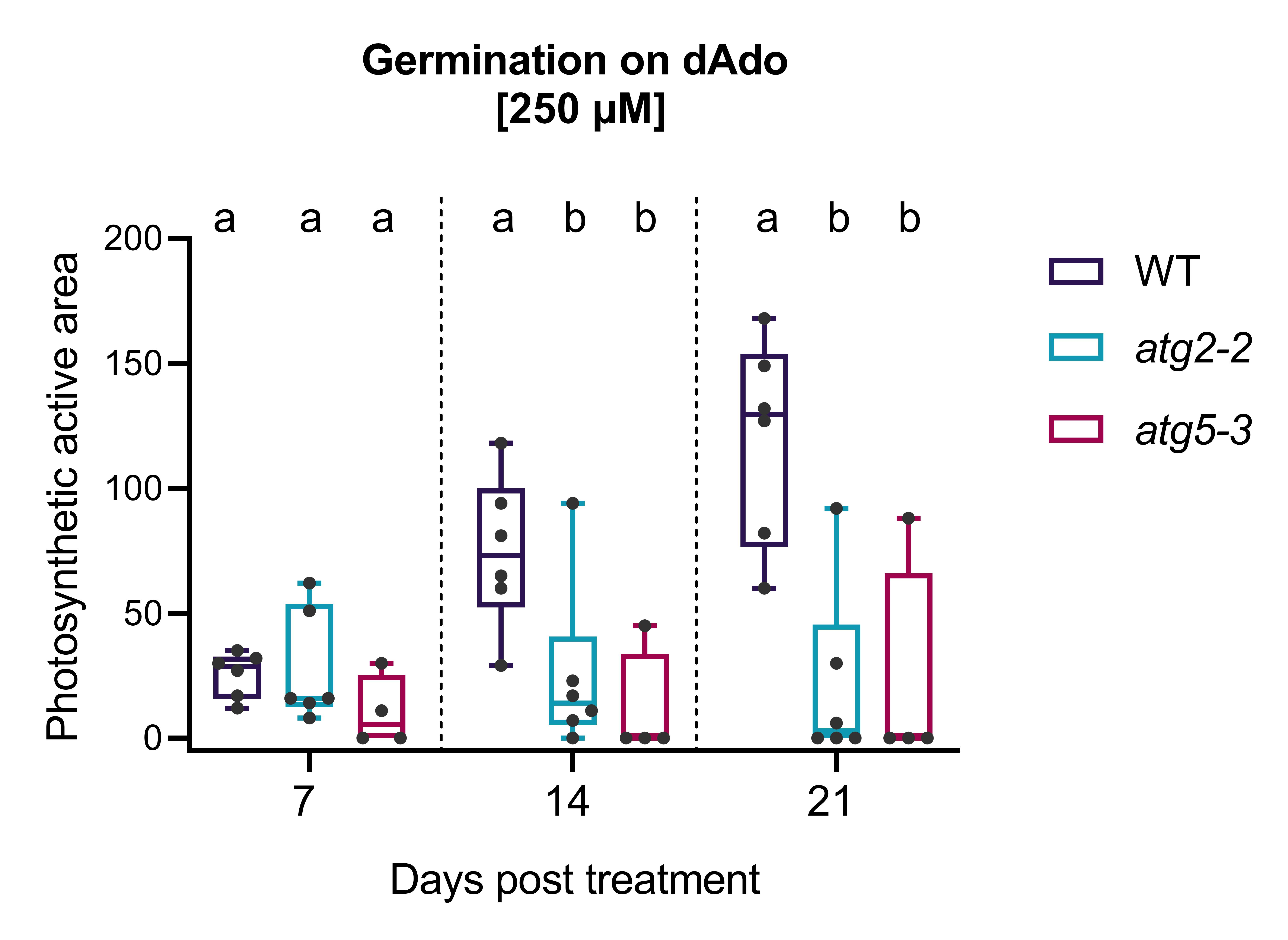


**Supplementary Figure S5: Seed germination assay in *A. thaliana* WT and autophagy mutants *atg5-3* and *atg2-2*.**

Quantification of photosynthetically active area at 7, 14, and 21 days of growth. Surface-sterilized seeds were transferred onto 1/10 PNM medium containing either mock (MES 2.5 mM buffer) or deoxyadenosine (dAdo, 250 µM). The box extends from the 25th to 75th percentiles, the center line is the median and the whiskers extend to the minimum and maximum values. Boxplots represent relative values from 6 independent biological replicates. Different letters indicate statistically significant differences (p < 0.05; one-way ANOVA with Tukey’s HSD post-hoc test).


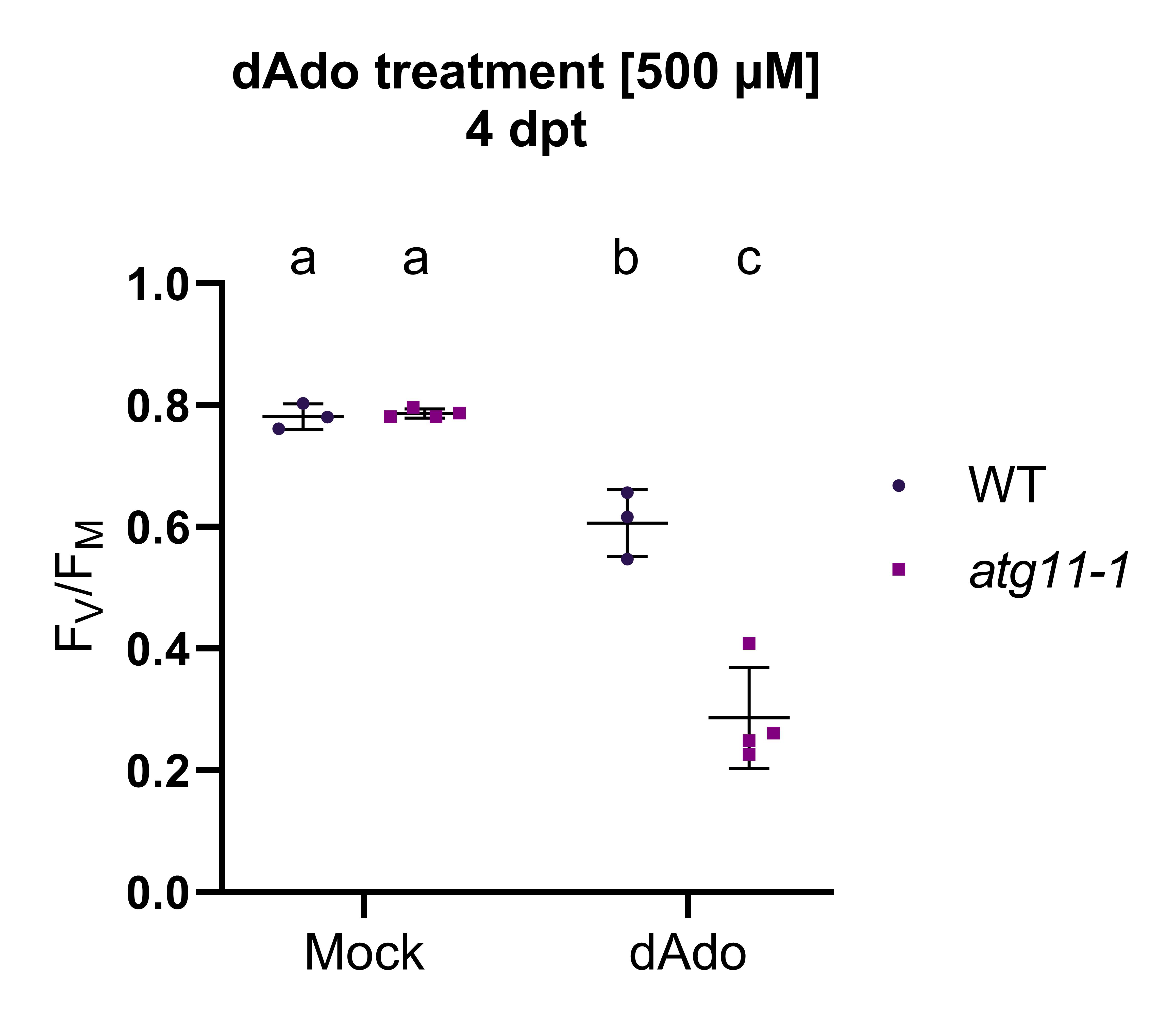


**Supplementary Figure S6: Autophagy mutant *atg11-1* show increased sensitivity upon dAdo treatment.**

Quantification of photosystem II maximum quantum yield (F_V_/F_M_) in WT and *atg11-1* seedlings at 4 days dpt (days post treatment), measured by PAM (Pulse amplitude modulation) fluorometry. Nine-day-old seedlings were treated with mock (MES 2.5 mM buffer) or deoxyadenosine (dAdo, 500 µM). The plot (mean ± SD) represents data from 3–4 independent biological replicates, each consisting of 12 wells with 3 seedlings per well. Different letters indicate statistically significant differences (p < 0.05; one-way ANOVA with Tukey’s HSD post-hoc test).


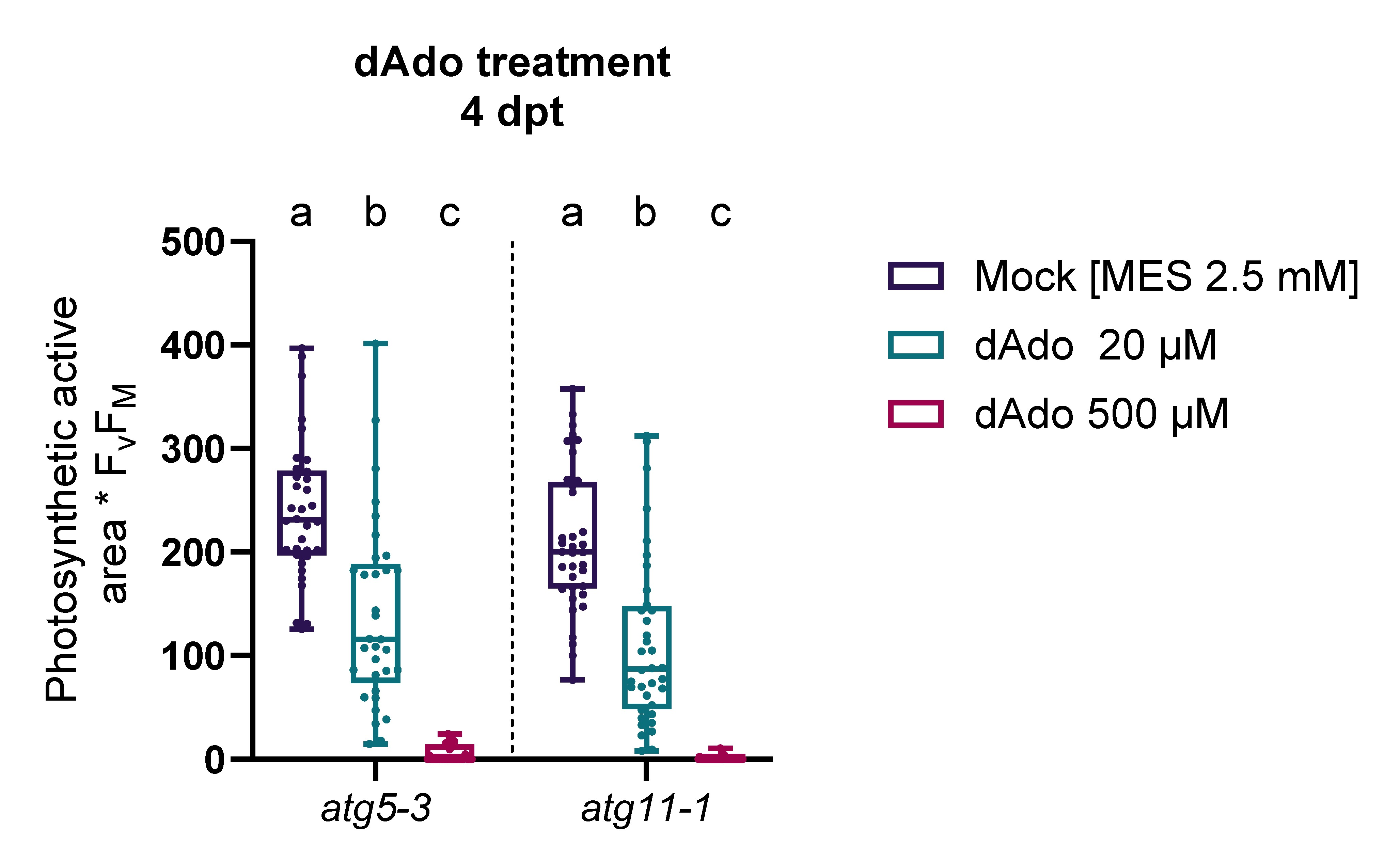


**Supplementary Figure S7: dAdo dose-dependent response in autophagy mutants.**

Quantification of photosystem II maximum quantum yield (F_V_/F_M_) in *atg5-3* and *atg11-1* seedlings at 4 dpt, measured by PAM (Pulse amplitude modulation) fluorometry. Nine-day-old seedlings were treated with mock (MES 2.5 mM buffer), dAdo (deoxyadenosine) (20 µM), or dAdo (500 µM). The box extends from the 25th to 75th percentiles, the center line is the median and the whiskers extend to the minimum and maximum values.Boxplots represent relative values from 33–36 biological replicates. Different letters indicate statistically significant differences (p < 0.05; one-way ANOVA with Tukey’s HSD post-hoc test).


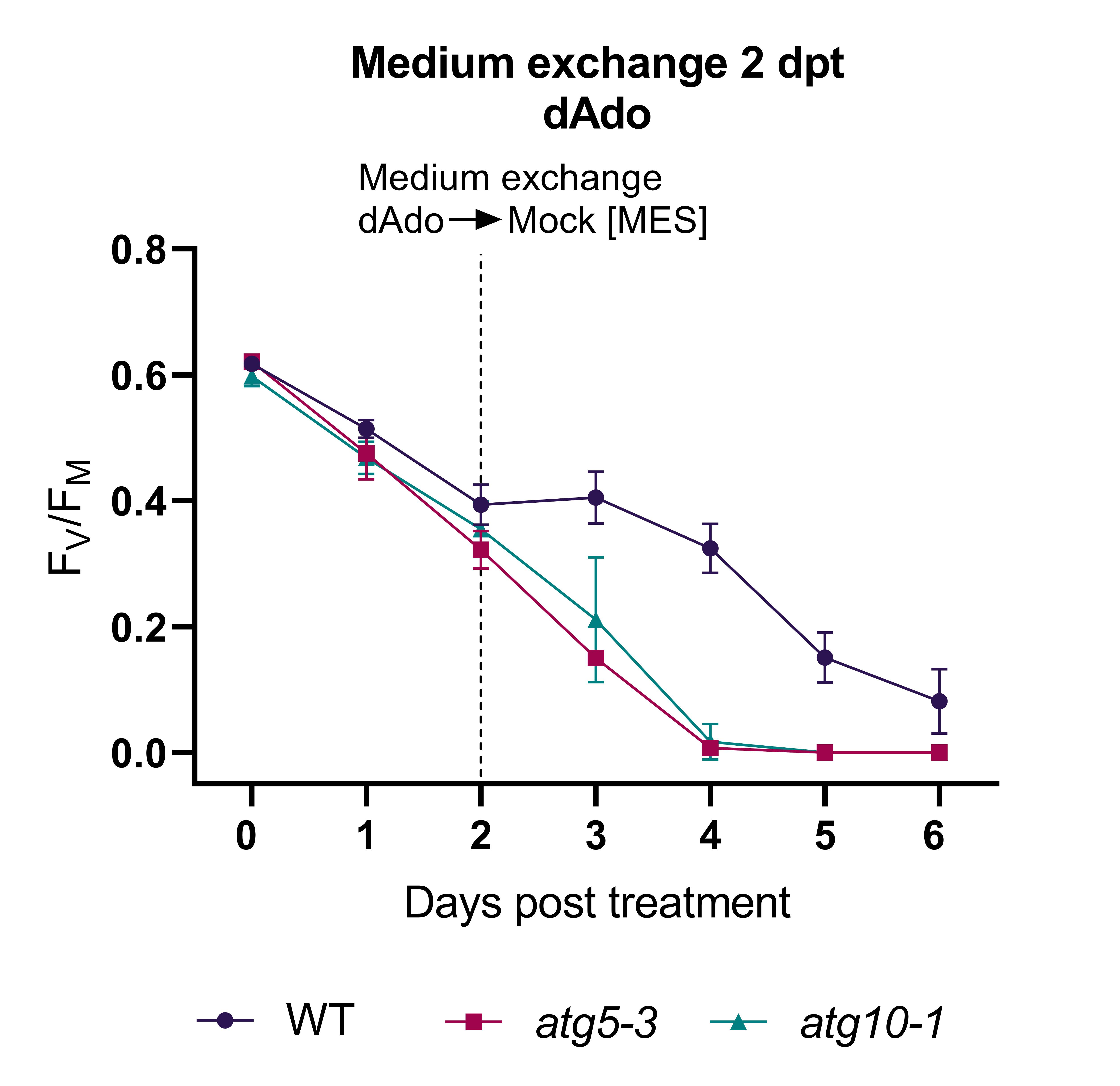


**Supplementary Figure S8: dAdo recovery assay in *A. thaliana* WT and autophagy mutants *atg5-3* and *atg10-1*.**

Photosystem II maximum quantum yield (F_V_/F_M_) measured in nine-day-old seedlings treated with either mock (MES 2.5 mM buffer) or deoxyadenosine (dAdo, 500 µM), using PAM (Pulse amplitude modulation) fluorometry. After 48 hours, the dAdo solution was replaced with fresh MES buffer, and recovery was monitored. Error bars represent ± SD of the mean from 3 independent biological replicates, each consisting of 12 wells with 3 seedlings per well.


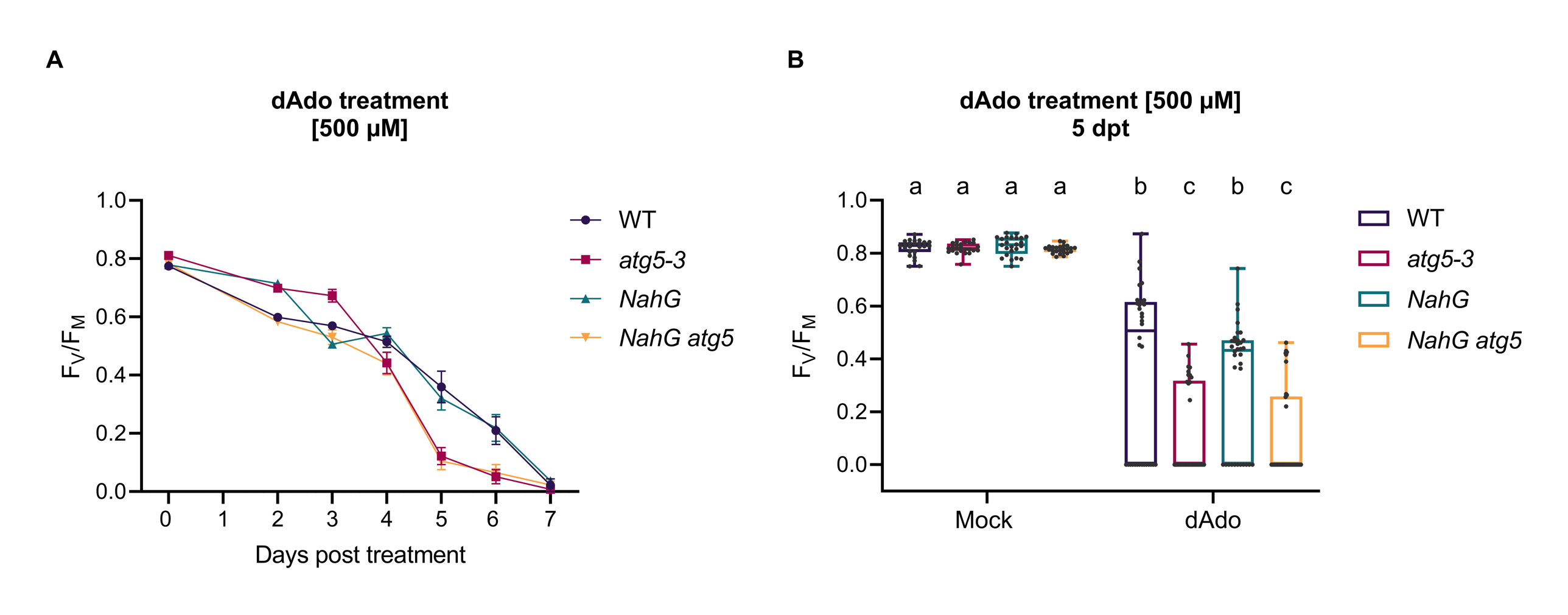


**Supplementary Figure S9: Increased sensitivity to dAdo treatment in *atg5* mutants is independent of SA accumulation.**

(**A**) Photosystem II maximum quantum yield (F_V_/F_M_) in nine-day-old seedlings treated with mock (MES 2.5 mM buffer) or deoxyadenosine (dAdo, 500 µM), measured by PAM (Pulse amplitude modulation) fluorometry. Error bars represent ± SEM of the mean from 34 independent biological replicates.
(**B**) Quantification of F_V_/F_M_ in WT, *NahG*, *atg5-3*, and *NahG* *atg5* seedlings at 5 dpt (days post treatment) with mock or deoxyadenosine (dAdo, 500 µM). The box extends from the 25th to 75th percentiles, the center line is the median and the whiskers extend to the minimum and maximum values. Boxplots represent relative data from 24–34 biological replicates. Different letters indicate statistically significant differences (p < 0.05; one-way ANOVA with Tukey’s HSD post-hoc test).

**Supplemental references**

Eichfeld R, Mahdi LK, De Quattro C, Armbruster L, Endeshaw AB, Miyauchi S, Hellmann MJ, Cord-Landwehr S, Peterson D, Singan V, Lail K, Savage E, Ng V, Grigoriev IV, Langen G, Moerschbacher BM, Zuccaro A (2024). Transcriptomics reveal a mechanism of niche defense: two beneficial root endophytes deploy an antimicrobial GH18-CBM5 chitinase to protect their hosts. New Phytol 244(3): 980-996.

Zuccaro A, Langen G (2020). Host-specific regulation of effector gene expression in mutualistic root endophytic fungi (Proposal ID: 505829). JGI Award DOI: 10.46936/10.25585/60001292.
